# Editorial: Restoring neural circuits after spinal cord injury

**DOI:** 10.3389/fnmol.2024.1428164

**Published:** 2024-06-17

**Authors:** Aikeremujiang Muheremu, Jianjun Wu

**Affiliations:** ^1^Key Laboratory of Orthopedic Regenerative Medicine, Sixth Affiliated Hospital of Xinjiang Medical University, Urumqi, China; ^2^Beijing Darwin Cell Biotechnology Co., Ltd., Beijing, China; ^3^Department of Spine Surgery, Fuzhou Second General Hospital, Fuzhou, Fujian, China; ^4^The School of Clinical Medicine, Fujian Medical University, Fuzhou, Fujian, China

**Keywords:** spinal cord injury, neural circuits, glial scar, axonal regeneration, neural repair

Although there are reports of restoring neural circuits to treat spinal cord injury, the results are not ideal. Lots of work needs to be done to find effective pharmaceutical, cellular, and tissue engineering approaches to restore neural circuitry after spinal cord injury, as well as their working mechanisms. In this Research Topic, we invited leading experts on spinal cord injury research to review their findings, ranging from the development of neural circuits in the spinal cord to various techniques in restoring those circuits to achieve functional recovery after spinal cord injury.

Spinal cord injury (SCI) leads to dysfunction in the spinal cord at and below the site of injury, resulting in impairments such as paralysis of movement and sensation. Depending on the severity of the injury, spontaneous recovery is often limited, leading to significant long-term consequences. In 2019, there were ~20.6 million individuals with SCI worldwide, with an incidence of 0.9 million new cases. The global age-standardized rates for SCI per 100,000 population in 2019 were 11.5 for incidence and 253.0 for prevalence (Safdarian et al., 2023). The indirect costs of SCI for each patient range from 0.5 to 2.3 million US dollars in the United States, 1.1 million pounds in the UK, and 0.7 to 1.3 million Canadian dollars in Canada (Cao and Krause, 2021), highlighting the enormous socio-economic impact of SCI.

During SCI, the primary injury triggers the production of free radicals, leading to a chronic state of ischemia and hypoxia. This results in glutamate excitotoxicity, lipid peroxidation, calcium influx, edema, and cellular damage. Subsequently, inflammation and immune responses compromise the integrity of adjacent tissues. Secondary injury processes, including demyelination of axons, glial cell proliferation, loss of damaged cells, and the disconnection of living neurons, create an environment that is not conducive to nerve regeneration, making it necessary to rebuild neural circuits to reconnect neurons on either side of the lesion (Hellenbrand et al., 2024).

Neural circuits exist in the spinal cord throughout an individual's life and are crucial for recovering locomotor function below the level of injury. To promote functional recovery, it is essential to enhance neuroplasticity, encourage the growth of injured and spared axons, strengthen remaining connections, and facilitate the formation of new neural circuits. However, the molecular mechanisms underlying the development of neural circuits in the spinal cord remain unclear. In the past decade, researchers have been investigating the mechanisms of spontaneous circuit reorganization and functional recovery after SCI. Promising approaches for regenerating impaired tissue and reviving dormant neural pathways include stem cell therapies, growth factors, novel biomaterials, microRNAs, and traditional herbal medicine (Gao et al., 2021; Bydon et al., 2024; Fang, 2024; Liu et al., 2024).

Several types of stem cells, including mesenchymal stem cells (MSCs), embryonic stem cells (ESCs), and induced pluripotent stem cells (iPSCs), are being explored for treating SCI. iPSCs, in particular, have the potential to differentiate into neurons, astrocytes, and oligodendrocytes, replacing damaged cells, regulating the spinal cord microenvironment, and promoting axon and myelin sheath regeneration (Hosseini et al., 2024). This leads to the reconstruction of neural circuits and facilitates functional recovery. Galiakberova et al. conducted a study using four distinct iPSC lines to create neural stem cells (NSCs) that spontaneously differentiated into neural cultures. Techniques such as immunocytochemistry, qPCR, bulk transcriptomics, and single-cell RNA sequencing at various stages showed that different NSC lines produce unique types of differentiated neural cells, crucial for optimal NSC development in SCI treatment. However, the propensity of NSCs to differentiate into astrocytes poses a challenge. To overcome this, strategies such as gene modification, the application of nanomaterials, and the use of ES cells are being considered. These findings underscore the need for further research into factors affecting cell stability in research settings (Hosseini et al., 2024).

In the context of spinal cord injury repair, gene expression plays a significant role in regulation. MicroRNAs (miRNAs), which are non-coding RNA molecules, influence mRNA expression to control protein synthesis. MicroRNAs have emerged as critical regulators in the molecular landscape of neural repair, especially following SCI. They promote functional recovery after SCI by modulating inflammatory responses (e.g., miR-155, miR-21), promoting neuronal survival and neuroprotection (e.g., miR-133b, miR-219), enhancing axonal regrowth and plasticity (e.g., miR-124, miR-29b), and facilitating myelin repair. Zhang et al., in a narrative review, highlighted the role of miRNAs in secondary damage mechanisms post-SCI, such as oxidative stress, apoptosis, autophagy, and inflammation, suggesting their potential as biomarkers for clinical diagnoses and therapeutic targets following SCI. Despite this potential, much work remains to develop effective delivery mechanisms that specifically target miRNAs to the injury site, determine the optimal timing for miRNA intervention, and understand the long-term impacts and potential side effects of miRNA-based therapies.

The application of Chinese herbal medicine (CHM) in the treatment of spinal cord injuries (SCI) and the restoration of neural circuits represents an intriguing area of interdisciplinary research that combines traditional medicinal practices with modern neuroscience. Traditionally, CHM has been used for thousands of years to treat various ailments, with a strong emphasis on promoting body balance and holistic recovery. In cases of spinal cord injuries, which often result in substantial and permanent impairments due to interrupted neural pathways, CHM potentially offers mechanisms to support neural recovery and functional improvement. Research has focused on several specific herbs and compounds, such as Ginseng, Dang Gui, and Gou Qizi, for their effectiveness in addressing neural damage and SCI. The mechanisms through which CHM aids in SCI recovery are diverse, often depending on specific herbs and their active components. In a study by Jia et al., tanshinone IIA, a lipophilic component of Danshen, is hypothesized to boost neuron survival and accelerate axonal repair after SCI due to its anti-inflammatory, antioxidant, and anti-apoptotic effects in the spinal cord. While this research sheds light on the potential mechanisms of tanshinone IIA in restoring neural pathways post-SCI, further evidence is required to confirm these hypotheses and explore their molecular mechanisms in-depth (Gao et al., 2021).

SCI primarily affects the spinal cord but also induces significant secondary changes in the brain, which can have profound implications for recovery and rehabilitation. The significance of morphological changes in the brain following SCI is a critical area of investigation, assisting researchers and clinicians in understanding the consequences of SCI on the central nervous system (CNS) and designing effective interventions. After SCI, the brain undergoes plastic changes as it adapts to the loss of sensory input and motor control. This plasticity involves the reorganization of neural circuits, particularly in areas directly connected to the injured segments of the spinal cord. However, while extensive research on SCI treatments has been conducted, morphological changes in the brain post-SCI are not fully understood regarding shifts in brain network hubs. In a study by Matsubayashi et al., resting-state functional magnetic resonance imaging (rsfMRI) revealed that the primary and secondary motor cortex holds high centrality post-injury, signifying their importance in motor function. Over time, centrality in the external capsule and putamen increases, indicating the dominance of the extrapyramidal/subcortical system. This study provides foundational insights into brain network changes post-SCI, aiding in the selection and evaluation of treatment efficacy for SCI patients (Lorach et al., 2023).

Despite significant progress in various research fields, such as inflammation, scar formation, cell transplantation, axon regeneration, and biomaterials-based repair, there are still no effective treatments to completely regenerate axons and rebuild neural circuits after SCI. The complexity of the pathological processes following SCI requires combinatorial strategies to effectively address the different aspects of the problem and achieve better functional recovery. Combinatorial therapies, which combine biomaterials, stem cells, growth factors, drugs, and exosomes, have shown promising results in promoting axon regeneration and the restoration of neural circuitry (Figure 1).

**Figure 1:**
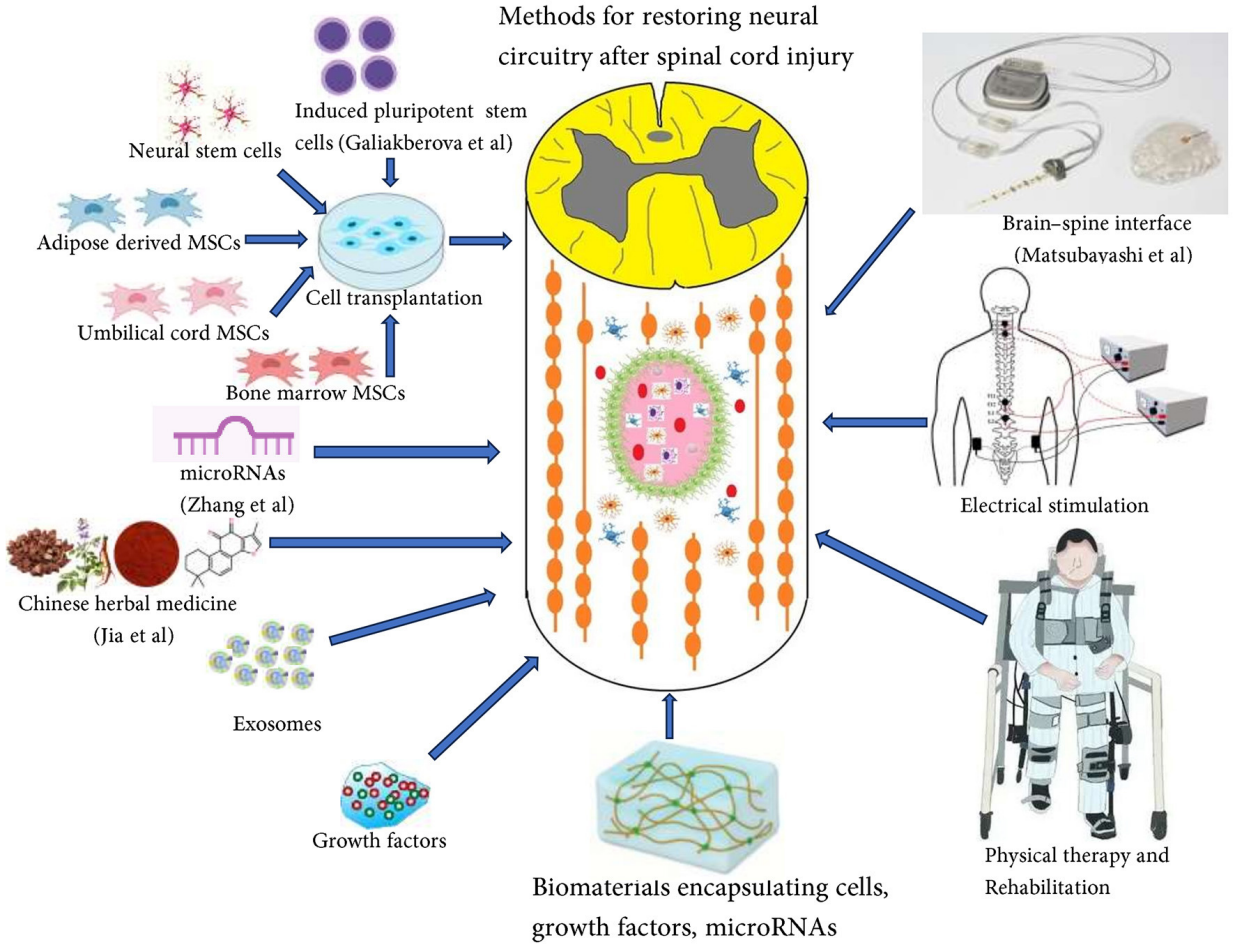
Combinatorial therapies including biomaterials, stem cells, growth factors, drugs, and exosomes, have shown promising results in promoting axon regeneration and restoration of neural circuitry.

It's important to note that axon regeneration alone is not sufficient for meaningful functional recovery. Electrical stimulation and digital bridging utilizing cortical implants between the brain and spinal cord show potential in reconstructing neural pathways and restoring functional recovery in individuals with spinal cord injuries (Lorach et al., 2023). Rehabilitation exercises continue to play a crucial role in the formation and remodeling of functional neural circuits, involving physical therapy, occupational therapy, and psychological support, enabling patients to regain autonomy and quality of life (He et al., 2024). The future of neural circuit restoration post-SCI shows promise. With each scientific advancement, we edge closer to reshaping the narrative of paralysis.

## Author contributions

AM: Conceptualization, Formal analysis, Investigation, Methodology, Writing – original draft, Writing – review & editing. JW: Conceptualization, Funding acquisition, Methodology, Resources, Supervision, Validation, Writing – original draft, Writing – review & editing.
